# Ligand-Enhanced Neodymium Doping of Perovskite Quantum Dots for Superior Exciton Confinement

**DOI:** 10.3390/ma16247585

**Published:** 2023-12-10

**Authors:** Xianghua Wang, Lin Zhou, Xudong Zhao, Wenlong Ma, Xinjun Wang

**Affiliations:** 1 Special Display and Imaging Technology Innovation Center of Anhui Province, Academy of Opto-Electric Technology, School of Instrument Science and Optoelectronics Engineering, Hefei University of Technology, Hefei 230009, China; 2021170058@mail.hfut.edu.cn (L.Z.); xudongzhao1998@outlook.com (X.Z.); 2021170050@mail.hfut.edu.cn (W.M.); wxj793949967@outlook.com (X.W.); 2 Anhui Province Key Laboratory of Measuring Theory and Precision Instrument, School of Instrument Science and Optoelectronics Engineering, Hefei University of Technology, Hefei 230009, China

**Keywords:** exciton, quantum dots, exciton binding energy, zeta potential, Förster resonance energy transfer, nanostrain, color conversion layer

## Abstract

In this study, all-inorganic perovskite quantum dots (QDs) for pure blue emission are explored for full-color displays. We prepared CsPbBr_3_ and Cs_3_NdCl_6_ QDs via hot injection methods and mixed in various ratios at room temperature for color blending. Nd-doped CsPb(Cl/Br)_3_ QDs showed a blueshift in emission, and the photoluminescence quantum yields (PLQY, Φ_PL_) were lower in the 460–470 nm range due to surface halogen and Cs vacancies. To address this, we introduced a silane molecule, APTMS, via a ligand exchange process, effectively repairing these vacancies and enhancing Nd doping into the lattice. This modification promotes the PLQY to 94% at 466 nm. Furthermore, combining these QDs with [1]Benzothieno[3,2-b][1]benzothiophene (BTBT), a conjugated small-molecule semiconductor, in a composite film reduced PLQY loss caused by FRET in solid-state QD films. This approach achieved a wide color gamut of 124% National Television System Committee (NTSC), using a UV LED backlight and RGB perovskite QDs in a BTBT-based organic matrix as the color conversion layer. Significantly, the photostability of this composite was enhanced when used as a color conversion layer (CCL) under blue-LED excitation.

## 1. Introduction

All-inorganic cesium lead halide perovskite (LHP) quantum dots (QDs), with the chemical formula CsPbX_3_ (where X represents Cl, Br or I), exhibit remarkable optical properties. They possess substantial absorption coefficients, high photoluminescent quantum yields (Φ_PL_), narrow emission bandwidths, and tunable emission peaks within the visible spectrum. These attributes render them highly suitable for a wide range of display applications, including energy-efficient smartphones, televisions, and various commercial display systems [1,2,3]. 

CsPbX_3_ has been developed as a color conversion phosphor to achieve a broader color gamut [4]. Compared to traditional blue GaN-LEDs, CsPbX_3_ with Cl/Br halide mixing offers superior color purity, characterized by a typical full-width at half-maximum (FWHM) of ~100 meV. This results in less harm to human eyes from overexposure to high-energy photons in wavelengths shorter than 450 nm. Higher Cl/Br ratios in the alloy or a quantum confinement effect can produce the desired blue emission, but these approaches might reduce Φ_PL_ due to increased surface and intrinsic defects with higher Cl content in the perovskite and a higher density of surface states in tightly confined perovskite QDs (PQDs) [5,6]. To improve the optical properties of blue LHP QDs, divalent or trivalent metal ions with smaller ionic radii than Pb^2+^ are introduced to partially substitute the B-site Pb^2+^ in LHP QDs. This incorporation enhances the coordination environment of Pb by reducing chlorine vacancy [7] and increasing lattice order [8]. While ion exchange effectively repairs intrinsic defects and optimizes lattice strain in the perovskite, surface defects require passivation with stronger ligands than the original oleic acid (OA) and Oleylamine (OAm) to enhance the thermal and photostability of the PQDs. 

The instability of QDs, evidenced by the degradation of their luminescence, is primarily caused by the chemical environment—specifically, the presence of oxygen and moisture—or exposure to light and heat [9]. In practice, most QD deterioration is the result of a combination of chemical, photonic, and thermal factors. Although atmospheric oxygen and moisture can be minimized through encapsulation, the photoluminescence of QDs must inherently withstand light exposure [10,11].

Intrinsic stability can be compromised by unstable surface ligand bonds, while defects in the QDs’ core lattice may hasten degradation [12]. Various strategies have been suggested to address these issues, including the introduction of functional additives [13,14,15], modification of atomic composition [14,16,17], and establishment of a surface passivation layer [18,19]. A recent technique involving surface passivation through ligand exchange with 3-aminopropyltrimethoxysilane (APTMS) has proven particularly effective at repairing cesium vacancies on the surface of perovskite QDs, significantly improving their photostability [13]. Nevertheless, reabsorption and Förster resonance energy transfer (FRET) are prevalent in solid QD films; both can diminish the photoluminescence quantum yield (Φ_PL_) compared to that of dilute QD solutions [20,21].

In this study, we synthesized intrinsically stable CsPbBr_3_ perovskite quantum dots (PQDs) by repairing Br and Cs vacancies through APTMS passivation. Following a documented procedure [14], these green CsPbBr3 PQDs were then combined with Cs_3_NdCl_6_ nanocrystals for neodymium and chlorine doping, resulting in blue PQDs termed Nd-CsPb(Cl/Br)_3_. Both Nd and Cl incorporation result in a photoluminescence (PL) blueshift. Notably, APTMS passivation facilitates efficient Nd doping into the PQDs. Additionally, the resultant favorable lattice strain and reduced dislocation density from Nd and chlorine incorporation significantly enhance the photostability of the perovskite structure, achieving a PLQY of up to 94% at 466 nm. These PQDs are well-suited for color conversion in light-emitting diodes (LEDs), as Nd doping lowers the exciton binding energy (EBE), thereby suppressing nonradiative FRET. Furthermore, embedding the PQDs within a matrix of BTBT, a conjugated small molecule, results in enhanced photostability of the color conversion layer.

## 2. Materials and Methods

Chemicals: Cesium carbonate (Cs_2_CO_3_), lead bromide (PbBr_2_, 99%), and lead chloride (PbCl_2_) were purchased from J&K Scientific. Oleylamine (OAm, 90%), oleic acid (OA, 90%), octadecene (ODE, 90%), and neodymium chloride (NdCl_3_, 99%) were purchased from Aladdin.

Preparation of Cs-Oleate: Cs_2_CO_3_ (0.407 g) was loaded into a 50 mL three-necked flask along with ODE (20 mL) and OA (1.25 mL), dried under vacuum for 1 h at 120 °C, and then heated under N_2_ at 150 °C until all Cs_2_CO_3_ reacted with OA. The solution was stored in a glass bottle and heated at 120 °C for 10 min to fully dissolve it before use.

Preparation of CsPbBr_3_ PQDs: To produce CsPbBr_3_ nanocrystals, a mixture of PbBr_2_ (0.2 g) and ODE (20 mL) was placed in a 50 mL three-necked flask and subjected to drying under vacuum conditions for one hour at a temperature of 120 °C. Subsequently, the atmosphere within the flask was replaced with nitrogen gas and maintained at the same temperature. Concurrently, pre-dried OAm (2 mL) and OA (2 mL) were introduced to the flask in a sequential manner. Following a 10 min period, the flask’s contents were subjected to a temperature increase up to 165 °C and maintained at that level for an additional 5 min. Next, Cs-oleate solution (2 mL) was rapidly injected into the mixture, and a brief 5 s later, the reaction was abruptly halted by immersing the flask in an ice water bath to cool the contents. The CsPbBr_3_ nanocrystals were then isolated from the resulting mixture by centrifugation, followed by a washing step using methyl acetate at a ratio of 1:2 to ensure thorough cleaning. Finally, for the purpose of purification, the nanocrystals were redissolved in n-hexane (10 mL), and the mixture was centrifuged at a speed of 5000 rpm for 3 min, after which the clear supernatant was removed, leaving behind the purified nanocrystals.

Synthesis of ExAP-CsPbBr_3_ NCs: For the synthesis of ExAP-CsPbBr_3_, APTMS was dropwise added into 5 mL CsPbBr_3_ (10 mg/mL) NCs dispersion according to specified concentrations and stirred for 30 min. Methyl acetate was then added to the NCs dispersion at a 2:1 volume ratio and centrifuged at 8000 rpm for 3 min. After centrifugation, the supernatant was discarded, and the precipitate was re-dispersed in n-hexane. The final concentration of the ExAP-CsPbBr_3_ NCs dispersed in n-hexane is 10 mg/mL.

Synthesis of CsPbCl_3_ NCs: For the synthesis of CsPbCl_3_ NCs, PbCl_2_ (0.105 g), ODE (10 mL), were added into a 50 mL three-neck, round-bottomed flask, dried under vacuum for 1 h at 120 °C, and then heated under N_2_ at 120 °C. Meanwhile, dried OAm (1 mL) and dried OA (1 mL) were subsequently injected into the mixed solution. After 10 min, the temperature was raised to 180 °C and kept at this temperature for 5 min. Then, the Cs-oleate solution (0.8 mL) was quickly injected, and 30 s later the reactant was cooled by an ice water bath. The CsPbCl_3_ NCs were extracted from the crude solution by centrifuging at 8500 rpm for 5 min to discard the supernatant containing unreacted precursor and by-products. The separated precipitation was redispersed in 5 mL n-hexane, and centrifugated at 8500 rpm for 5 min. After removing the supernatant, the purified precipitation of CsPbCl_3_ NCs was dispersed in n-hexane.

Preparation of Cs_3_NdCl_6_ NCs: For the synthesis of Cs_3_NdCl_6_ NCs, NdCl_3_ (0.127 g) and ODE (20 mL) were added into a 50 mL three-neck, round-bottomed flask, dried under vacuum for 1 h at 120 °C, and then heated under N_2_ at 120 °C. Meanwhile, dried OAm (2 mL) and dried OA (2 mL) were subsequently injected into the mixed solution. After 10 min, the temperature was raised to 170 °C and kept at this temperature for 5 min. Then, the Cs-oleate solution (2 mL) was quickly injected, and 30 s later the reaction mixture was cooled by an ice water bath. The Cs_3_NdCl_6_ NCs were extracted from the crude solution by centrifuging at 8500 rpm for 5 min to discard the supernatant containing unreacted precursor and by-products. After that, 5 mL of n-hexane was added into the precipitates to disperse, and then the solution was centrifugated at 8500 rpm for 5 min. After centrifugation, the supernatant was discarded, and the precipitates were re-dispersed in n-hexane.

Preparation of CsPb(Br/Cl)_3_ solution: CsPbCl_3_ dispersion (10 mg/mL) was mixed with a CsPbBr_3_ dispersion (10 mg/mL) at different volume ratios, and kept stirring for 10 min to complete the solution reaction.

Preparation of Nd-CsPb(Cl/Br)_3_ solution: Cs_3_NdCl_6_ dispersion (10 mg/mL) was mixed with a CsPbBr_3_ dispersion (10 mg/mL) at different volume ratios, and kept stirring for 10 min to complete the solution reaction.

Preparation of Nd-ExAP-CsPb(Cl/Br)_3_ solution: Cs_3_NdCl_6_ dispersion (10 mg/mL) was mixed with an ExAP-CsPbBr_3_ dispersion (10 mg/mL) at different volume ratios, and kept stirring for 10 min to complete the solution reaction.

Synthesis of BTBT: For the synthesis of [1]Benzothieno[3,2-b][1]benzothiophene (BTBT), 35.6 mmol o-chlorobenzaldehyde (OCBA) and 71.2 mmol sodium hydrosulfide hydrate were added to 20 mL N-methylpyrrolidone (NMP); the temperature was raised to 80 °C and stirred for 1 h. Then, the mixture was heated to 190 °C, stirred at the same temperature for 10 h, and cooled to room temperature. The resulting mixture was poured into 100 mL aqueous solution of saturated ammonium chloride and cooled in an ice water bath. The sediment was collected through filtration and washed twice with water and four times with acetone to obtain crude yellow BTBT precipitate, which was further purified by recrystallization from toluene to obtain 1.5 g BTBT as a yellow-white solid.

Characterization: UV-Vis absorbance spectra of CsPbBr_3_ PeNCs were collected in transmission mode using a UV-2550 ultraviolet spectrophotometer (Shimadzu, Kyoto, Japan). The specimens for optical measurements were prepared at a concentration of 50 μg/mL and measured in a cuvette of 1 cm optical length. Emission spectra of CsPbBr_3_ PeNCs were collected (with excitation/emission slit width of 1 nm) by a spectrofluorometer (Fluoromax-4, Horiba, Kyoto, Japan), and the quantum yields were determined by using a Quanta-φ integrating sphere accessory (slit width = 1 nm). Relative PLQY were estimated using photoluminescence excitation spectra (PLE, with the intensity denoted by I_PLE_) and absorption (A) of the colloidal solution, and defined as I_PLE_/(1-1/10A). The morphology and structure of the CsPbBr_3_ PeNCs were characterized by using a transmission electron microscope (TEM, JEM-2100F, JEOL, Tokyo, Japan) and X-ray diffractometer (PANalytical X-Pert PRO MPD, PANalytical B.V., Almelo, Holland), respectively. The TEM and HRTEM images of the NCs were captured at 120 kV and 200 kV, respectively. Fourier infrared spectrum (FTIR) was performed using a Nicolet 6700 spectrometer (Thermo Fisher Scientific, Waltham, MA, USA). The XPS of CsPbBr_3_ PeNCs was measured with a photoelectric spectrometer (Escalab 250XI, Thermo Fisher Scientific, Waltham, MA, USA). 

The exciton binding energy is defined as the difference between the excitonic gap and the true band gap (i.e., without an exciton), and estimated according to the room temperature UV-Vis absorption of the QD solution using Elliott’s formula [22]. To evaluate the surface electrical properties of CsPbBr_3_ PeNCs, the Zeta potential was measured by the laser-Doppler electrophoresis method, using a Zeta potential Analyzer (Nano ZS90, Malvern Company, Malvern City, UK). Measurements were conducted in n-hexane at room temperature. The Zeta potential was calculated based on the following Henry Equation:

$$U_{E}=2\varepsilon \xi f(ka)/3\eta$$

where ξ is the Zeta potential, U_E_ is the electrophoretic mobility, η is the viscosity, ε is the permittivity of the medium, and f(ka) is Henry’s function.

## 3. Results and Discussion

**Figure 1:**
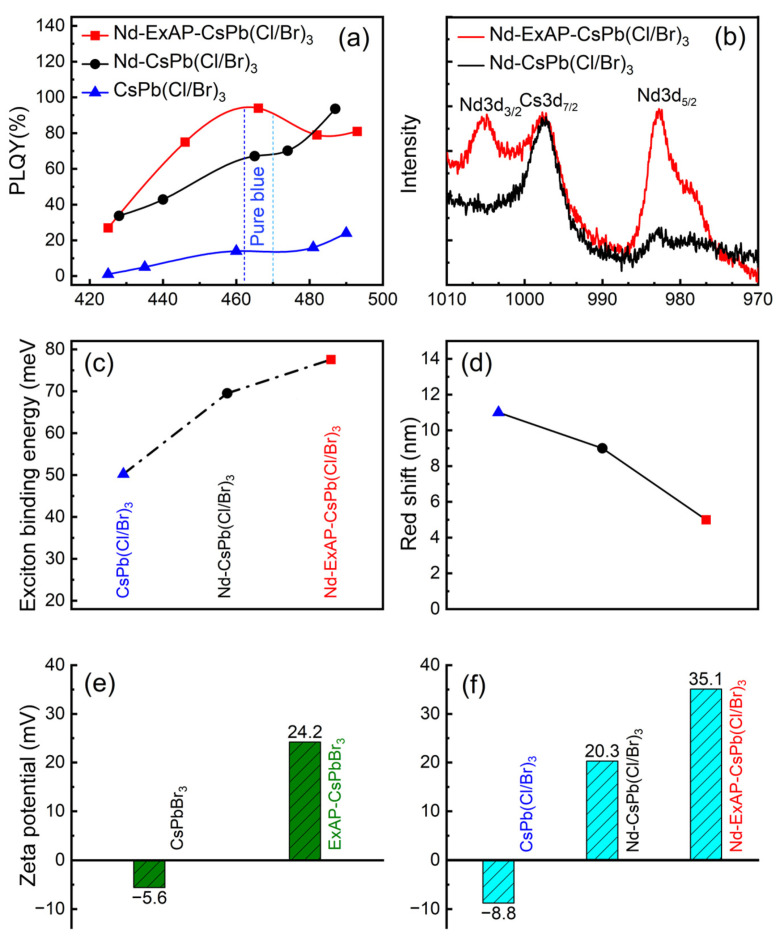
(**a**) Nd-doping Effect on Φ_PL_ via halide exchange, using ExAP-CsPbBr_3_ NCs (red) in comparison to using the pristine CsPbBr_3_ NCs (black). Baseline: halide exchange with CsPbCl_3_ NCs (blue). (**b**) High-resolution XPS of Nd 3d; Effect of Nd-doping on (**c**) exciton binding energy and (**d**) relative optical redshift of the solid-state PQD CCLs, with respect to their solution form; (**e**) Zeta potential of green CsPbBr_3_ PQDs and ExAP-CsPbBr_3_ PQDs; (**f**) Effect of Nd-doping on Zeta potential of blue PQDs.

**Figure 2:**
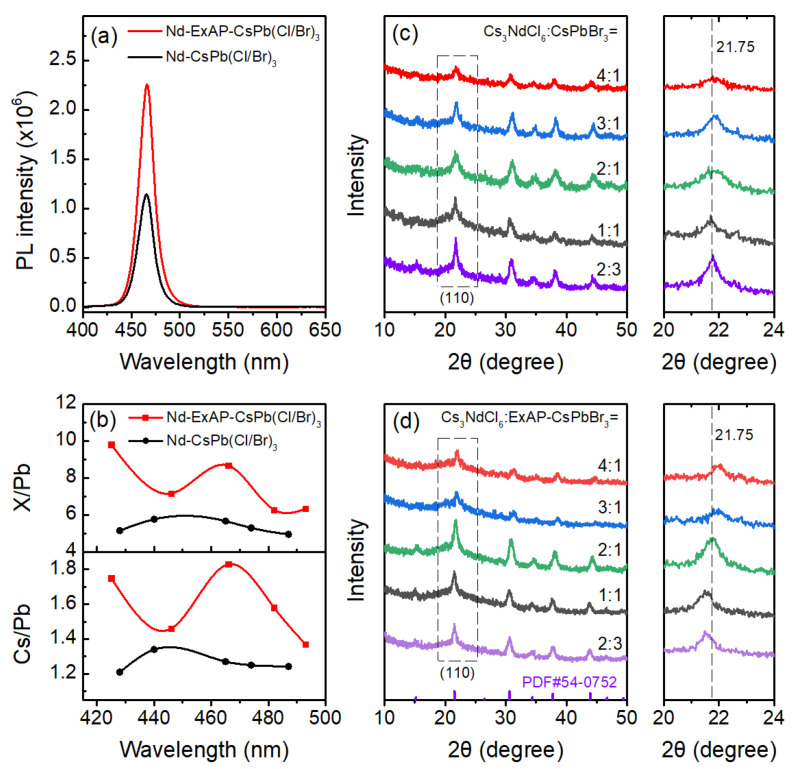
(**a**) PL spectra under 365 nm excitation and (**b**) Cs/Pb and X/Pb atomic ratios estimated by XPS; Nd-ExAP-CsPb(Cl/Br)_3_ (red), Nd-CsPb(Cl/Br)_3_ (black); (**c**,**d**) Evolution of their PXRD patterns with increasing Pb-site substitution by Nd. The diffraction peaks of (110) facets vary slightly in the 2θ angles, while the optimized strain states have a strong (110) diffraction precisely at 21.75°.

Both CsPbBr_3_ and Cs_3_NdCl_6_ were synthesized using the hot injection procedure. To obtain a widely tunable bandwidth of the blue QDs, APTMS-ligand-exchanged CsPbBr_3_ (ExAP-CsPbBr_3_) and the original CsPbBr_3_ were, respectively, mixed with the Cs_3_NdCl_6_ NCs at varying mixing ratios for halide exchange and Nd doping. Φ_PL_ was measured for resulting samples, denoted by Nd-ExAP-CsPb(Cl/Br)_3_ and Nd-CsPb(Cl/Br)_3_, respectively, as shown in Figure 1a in comparison with CsPb(Cl/Br)_3_ samples, which were synthesized via sole halide exchange between CsPbBr_3_ and CsPbCl_3_ [14]. The Φ_PL_ of Nd-CsPb(Cl/Br)_3_ is significantly enhanced in the spectral range of 420–490 nm, compared to CsPb(Cl/Br)_3_. Meanwhile, the photothermal stability of Nd-CsPb(Cl/Br)_3_ NCs is superior to CsPb(Cl/Br)_3_ NCs (Figure S1). Therefore, Nd doping notably promotes the brightness and intrinsic stability of PQDs. The Nd-CsPb(Cl/Br)_3_ powder obtained at a Cs_3_NdCl_6_:CsPbBr_3_ mixing ratio of 2:3 exhibited the strongest (110) diffraction at 21.75° in the powder X-ray diffraction (PXRD) as shown in Figure 2c, indicating a high crystallinity and stability of the perovskite structure. High-resolution XPS shows a weak peak of Nd 3d^5/2^ at 983 eV, signifying a 4.8% doping concentration (Figure 1b). This sample exhibits a sky blue emission with a Φ_PL_ maxima of 93% at 487 nm, but the Φ_PL_ of Nd-CsPb(Cl/Br)_3_ samples in the 460–470 nm pure blue band was lower than 70% (Figure 1a).

For weakly confined PQDs, the PXRD pattern identifies a tensile strain arising from the APTMS treatment at relatively low blending ratios. With the increase of Nd incorporation, the tensile strain is relieved. Moreover, the intensity and 2θ angle of the (110) diffraction in the XRD pattern clearly indicate a strain state that relates to a higher Φ_PL_ of the phosphor, characterized by a strong diffraction at 21.75°. In the case of Nd-ExAP-CsPb(Cl/Br)_3_ samples, the strongest (110) diffraction also appears at 21.75°, which corresponds to a Cs_3_NdCl_6_:ExAP-CsPbBr_3_ mixing ratio of 2:1; meanwhile, an Φ_PL_ maxima of 94% is achieved at 466 nm, which lies within the pure blue band. High brightness pure blue PQDs are synthesized using the color mixing procedure that blends ExAP-CsPbBr_3_ and Cs_3_NdCl_6_ for ion exchange and strain modulation in a single step. The optimal Φ_PL_ correlates with a favorable strain state, evidenced by a strong diffraction at 21.75° (Figure 2d). These pure blue PQDs exhibit intrinsic stability due to significantly reduced cesium and halogen vacancies (Figure 2b), making them well-suited for color conversion in lighting and display technologies. However, increasing the mixing ratio leads to a decrease in Φ_PL_ to below 30%, with a blue shift in emission to 425 nm. This trend may be induced by an unfavorable strain state in the PQDs, and increased short-range disorder [17] at higher Cl/Br ratios in the QDs. Given that the ionic radius of Nd^3+^ (98 pm) is substantially smaller than that of Pb^2+^ (119 pm) and the chloride ion is smaller than the bromide ion, increasing the proportion of Cs_3_NdCl_6_ relative to ExAP-CsPbBr_3_ shifts XRD peaks to higher 2θ angles.

In the high-resolution XPS spectra, the Nd 3d^5/2^ peak at 983 eV (Figure 1b) exhibits a marked difference in intensity between the two sets of Nd-doped samples, elucidating the impact of APTMS ligand exchange on cation exchange efficiency. The molar fraction of Nd in Nd-ExAP-CsPb(Cl/Br)_3_ is estimated to be 11.2%, significantly higher than the 4.8% estimated in Nd-CsPb(Cl/Br)_3_. This higher Nd doping level contributes to an increased exciton binding energy (EBE) in the QDs [23], enhancing brightness and mitigating FRET-induced PL redshift in QD films. The elevated EBE is confirmed by experimental data, as shown in Figure 1c, which were extracted by fitting the UV-Vis absorption of the QD solutions to the Elliott’s model (Figure S2). 

Moreover, excitons, being tightly bound, prefer monomolecular recombination over dissociation into free carriers susceptible to defect capture [24]. Additionally, both Nd doping and APTMS treatment result in positive surface charging, demonstrated by the higher Zeta potential readings in Figure 1e,f. The increased magnitude of the Zeta potential indicates enhanced stability of the QD solution.

**Figure 3:**
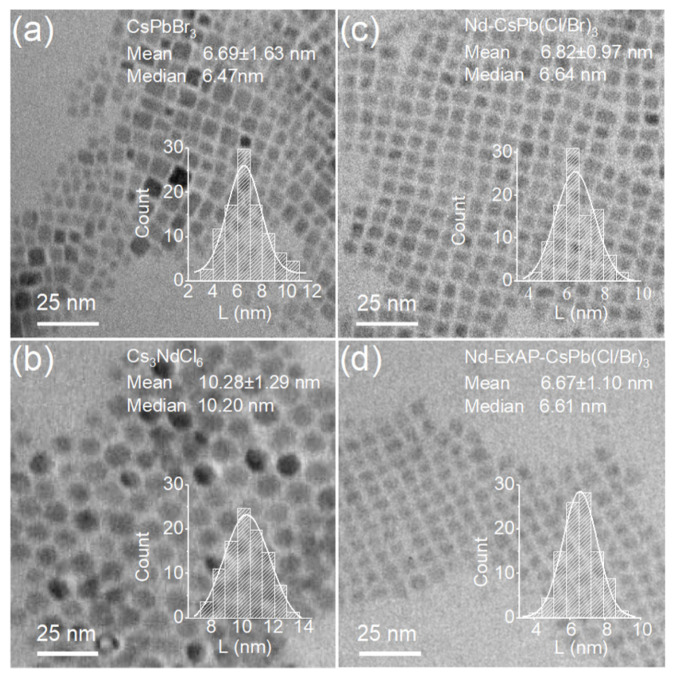
TEM images and statistics on size distribution for (**a**) pristine CsPbBr_3_ PeNCs, (**b**) Cs_3_NdCl_6_ NCs, and Nd-doped CPbBr_3_ PeNCs (**c**), without and (**d**) with an APTMS ligand exchange procedure. In each histogram inset, L represents the measured edge length of the cuboid-shaped nanocrystals, or the diameter of the spherical nanocrystals.

The pure blue photoluminescence (PL) spectra, exhibiting an emission peak at 466 nm, are depicted in Figure 2a. The PL intensity of Nd-ExAP-CsPb(Cl/Br)_3_ PQDs is twice as high as that of Nd-CsPb(Cl/Br)_3_. This enhancement is largely due to the substantial reduction in cesium and halide vacancies on the surface, a fact corroborated by the atomic ratios obtained from XPS analysis (Figure 2b). The highest brightness in Nd-ExAP-CsPb(Cl/Br)_3_ is achieved with a mixing ratio of Cs_3_NdCl_6_ to ExAP-CsPbBr3 of 2:1, triple the optimized ratio for Nd-CsPb(Cl/Br)_3_. It is therefore inferred that the former attains its optimal strain state with a greater level of Nd incorporation. In summary, APTMS passivation not only aids in repairing surface vacancies, as previously demonstrated [13], but also encourages Nd doping. Further, the significantly increased fraction of Nd leads to a notably higher exciton binding energy, which occurs despite the quantum dots being comparable in size, as illustrated by the TEM images shown in Figure 3.

The TEM image in Figure 3a reveals a broader size dispersion of pristine CsPbBr_3_ (6.69 ± 1.63 nm), which are used as intermediate QDs for color mixing. As depicted in Figure 3b, Cs_3_NdCl_6_ NCs serve as sources of chlorine and neodymium. Their dissolution during the ion exchange process creates a halogen-rich environment, contributing to a more uniform QD size [25]. As a result, the Nd-doped blue PQDs, showcased in Figure 3c,d, display a monodisperse morphology with narrow size distributions. This monodispersity, combined with a higher EBE, effectively suppresses FRET effects. FRET is essentially a resonance transfer [26] of electronic excitation resulting from the coupling between the transition dipoles of an excited donor and a ground state acceptor. In this context, high-energy emissions from smaller QDs (acting as donors) might be quenched by larger QDs (acting as acceptors) via FRET, leading to a red-shifted emission in the static PL of the assembly.

**Figure 4:**
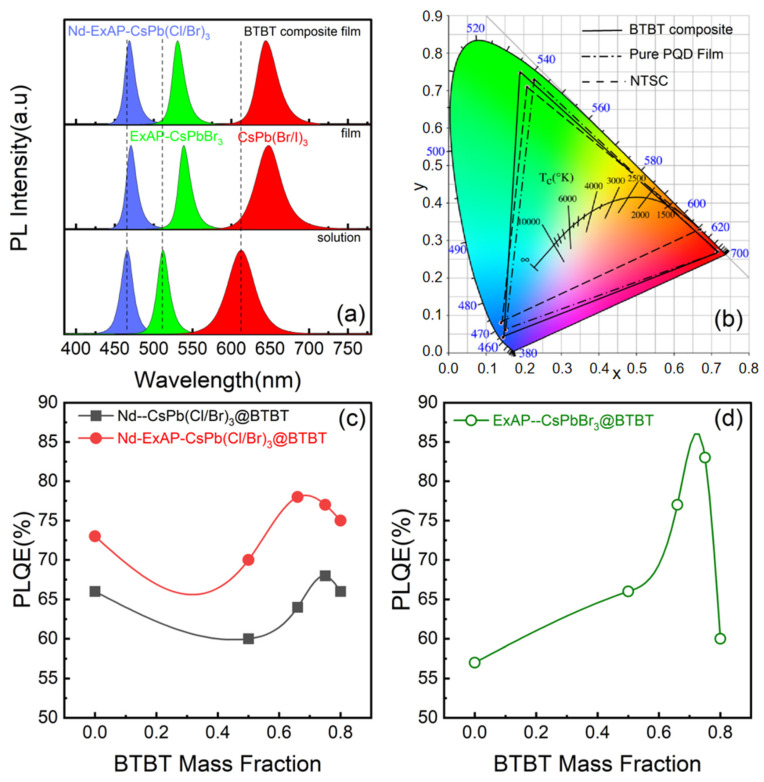
(**a**) Emission spectra of blue, green, and red PQDs in the form of solution (Bottom) and CCLs composed of pure PQDs (Middle), or PQD:BTBT composites (Top). All spectra are photoexcited by commercial UV GaN-LED chips. Blue: Nd-ExAP-CsPb(Cl/Br)_3_, Green: ExAP-CsPb(Cl/Br)_3_, Red: CsPb(Br/I)_3_. (**b**) CIE 1931 chromaticity coordinates of blue, green, and red LEDs with CCLs. (**c**,**d**) Evolution of PLQE under 365 nm UV excitation with increasing BTBT mass fraction of the (**c**) blue and (**c**) green composite CCLs.

A high exciton binding energy is crucially important for light-emitting devices, both in electroluminescence [27] and color conversion applications. Figure 4 presents the dry powder emission spectra of the blue, green, and red PQDs as the color conversion layer (CCL) on a 365 nm UV LED chip, along with their coordinates in the Commission Internationale de l’Eclairage (CIE) 1931 color space. The LED chip operates at an approximate bias voltage of 3.0 V and a constant forward current of 20 mA. The blue PQD CCL, composed of Nd-ExAP-CsPb(Cl/Br)_3_, shows a minimal red shift compared to the green and red CCLs, which are made of PQDs without Nd doping. Specifically, the PL peak of the green ExAP-CsPbBr_3_ CCL is red shifted from 512 nm (as measured in solution) to 539 nm, indicating a red shift of 27 nm. As shown in Table 1 and Figure 4, this red shift is significantly reduced for Nd-doped blue PQDs, with only a 5 nm shift from 466 to 471 nm, corresponding to a minor red shift of 28 meV in photon energy. For green and red CCLs, the emission peaks shift by over 100 meV. Since there is no correlation between the red shift in photon energy and the emission wavelength, the limited red shift observed in the blue CCL can be attributed to the higher exciton binding energy of the blue PQDs.

The FRET efficiency loss in conventional QDs can be effectively mitigated by engineering the shell thickness [28], or alternatively, tailored by embedding the QDs in an organic matrix [29]. In this study, BTBT composite CCLs were prepared by blending at a mass ratio of 1:1. Under 365 nm UV excitation, the blue, green, and red emission peaks of these composite CCLs are positioned at 469, 531, and 644 nm, respectively. Consequently, the red shift was reduced to 3, 19, and 31 nm, respectively. It is apparent that Nd^3+^ doping plays a more important role in reducing FRET efficiency than the organic matrix, likely due to the inadequate dielectric confinement [27]. Given that FRET is strongly distance dependent, its effects can be effectively nullified by sufficient dilution [30]. Hence, a consistent blue shift can be observed in the PL spectrum with increasing BTBT mass fraction (Figure S3). Partly because of the abated FRET loss, PLQEs of the blue and green composite films are optimized at a BTBT mass fraction of around 70% (Figure 4c,d).

PLQE enhancement in green CCLs is notably pronounced due to the smaller EBE of green PQDs. Therefore, it is deduced that FRET can be inhibited either with a higher EBE of the inorganic PQDs, or with sufficient dilution in an organic matrix. Furthermore, FRET has potential applications in advanced optoelectronics [31]. It is hypothesized that domain matching epitaxy between the perovskite and the organic lattice could induce a global strain field, presenting an additional mechanism for strain engineering that warrants further exploration. Additionally, the color gamut is enhanced as the y-coordinate of the green PQDs peaks at an optimized BTBT mass fraction. In the case of blue PQD composite films, both the x- and y-coordinates decrease with increasing BTBT mass fraction. Consequently, the color gamut achieved by PQD/BTBT composite CCLs reaches 124% of the National Television System Committee (NTSC) standard, surpassing the performance of pure PQD CCLs by 10%.

**Figure 5:**
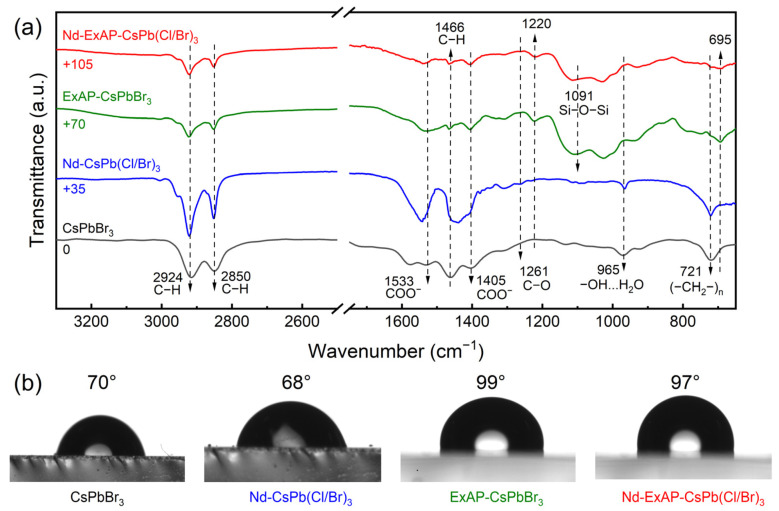
(**a**) Powder FTIR of the PQDs and (**b**) water contact angle measurements performed on the spin-coated thin films.

Last but not least, the intrinsic stability of Nd-ExAP-CsPb(Cl/Br)_3_ PQDs is superior to that of Nd-CsPb(Cl/Br)_3_, as evidenced by the stability tests conducted at 100 °C, or under UV exposure (Figure S4). This enhanced stability is attributed the increased formation energy of cesium vacancies in PQDs with APTMS bound to their surface [13]. FTIR spectra of Nd-CsPb(Cl/Br)_3_ PQDs reveal the presence of OA through characteristic vibrations at 1533 and 1405 cm^−1^ (Figure 5a), while a distinct peak at 965 cm^−1^ indicates a hydrophilic surface nature. These vibrational features are nearly eliminated following APTMS passivation, with siloxane bands emerging instead, leading to a hydrophobic surface as verified by water contact angle measurements (Figure 5b). Furthermore, the BTBT matrix itself is hydrophobic [32], exhibiting a water contact angle of 90.6°, which further enhances the ambient stability of the CCL component under blue-LED excitation (Figure S5).

## 4. Conclusions

In summary, treating CsPbBr_3_ PQDs with APTMS ligand exchange substantially improves their photostability and introduces a tensile strain, allowing for efficient Nd^3+^ ion exchange at the Pb site in quantities greater than 10%. This process yields bright, pure blue photoluminescence with a high PL quantum efficiency of 94% at 466 nm upon optimizing lattice strain. Moreover, Nd incorporation raises the exciton binding energy, thereby inhibiting FRET in the blue-emitting PQDs. When embedded in a BTBT organic matrix as color conversion layers for UV LEDs, the PQDs exhibit enhanced stability and better CIE coordinates by minimizing FRET effects and leveraging the organic lattice’s strain control. These PQDs are not only suited for academic investigations into quantum physics and nanomaterials, but are also promising components for next-gen electronic devices, such as micro-LEDs and QLEDs. Looking forward, ligand-facilitated ion exchange and strain engineering are expected to broaden the scope and integration of nanomaterials suitable for cost-efficient optoelectronics.

## Figures and Tables

**Table 1:** CCL emission peak wavelength in comparison with PQD solution and the red shift in meV.

PQDs Form	Blue	Green	Red
PQD solution	466	512	613
Pure PQD CCL	471 (28 meV)	539 (121 meV)	647 (106 meV)
PQD/BTBT Composite	469 (17 meV)	531 (87 meV)	644 (97 meV)

## Data Availability

Data is contained within the article or Supplementary Material.

## Supplementary Materials

The following supporting information can be downloaded at: https://www.mdpi.com/article/10.3390/ma16247585/s1, Figure S1. Effects of Nd-doping on (a–c) thermostability and (d–f) photostability of spin-coated films on glass slides. PL spectra and the relative intensity evolution over (a–c) a hotplate at 100 °C, or (d–f) under continuous irradiation with 365 nm UV lamp, Figure S2. The exciton binding energy was calculated using the Elliott’s model. Nd doping would increase exciton binding energy, Figure S3. Effect of the amount of BTBT on the red shift. FRET can be eliminated by sufficient dilution, Figure S4. Effects of surface passivation on (a–c) Thermal and (d–f) Photo stability of spin-coated films on glass slides. PL spectra and relative intensity evolution (a–c) under 100 °C heating or (d–f) continuous irradiation with 365 nm UV lamp, Figure S5. PL intensity of BTBT composite CCLs under prolonged exposure to blue LED backlight (in days); Left panel: green LED under 466 nm photoexcitation; Right panel: blue LED under 440 nm photoexcitation.
