# Supplementary material for: Ligand-Enhanced Neodymium Doping of Perovskite Quantum Dots for Superior Exciton Confinement
Source: Materials (Basel). 2023 Dec 10;16(24):7585. doi: 10.3390/ma16247585 (PMC10744661; doi:10.3390/ma16247585)
Supplement: Supplementary file 1 [file materials-16-07585-s001.zip › materials-2725500-supplementary.pdf]

materials

MDPI

Supporting information

Article

Ligand-Enhanced Neodymium Doping of Perovskite Quantum Dots for Superior Exciton Confinement

Xianghua Wang [1,2,*], Lin Zhou [1,2], Xudong Zhao [1,2], Wenlong Ma [1,2] and Xinjun Wang [1,2]

1 Special Display and Imaging Technology Innovation Center of Anhui Province, Academy of Opto-Electric Technology, School of Instrument Science and Optoelectronics Engineering, Hefei University of Technology, Hefei 230009, China; 2021170058@mail.hfut.edu.cn (L.Z.); xudongzhao1998@outlook.com (X.Z.); 2021170050@mail.hfut.edu.cn (W.M.); wxj793949967@outlook.com (X.W.)

2 Anhui Province Key Laboratory of Measuring Theory and Precision Instrument, School of Instrument Science and Optoelectronics Engineering, Hefei University of Technology, Hefei 230009, China

* Correspondence: xhwang@hfut.edu.cn

Citation: Wang, X.; Zhou, L.; Zhao, X.; Ma, W.; Wang, X. Ligand-Enhanced Neodymium Doping of Perovskite Quantum Dots for Superior Exciton Confinement. *Materials* **2023**, *16*, x. https://doi.org/10.3390/xxxxx

Academic Editor: Heesun Yang

Received: 2 November 2023
Revised: 30 November 2023
Accepted: 4 December 2023
Published: 6 December 2023

Copyright: © 2023 by the authors. Licensee MDPI, Basel, Switzerland. This article is an open access article distributed under the terms and conditions of the Creative Commons Attribution (CC BY) license (https://creativecommons.org/licenses/by/4.0/).

S1: Effects of Nd-doping on photothermal stability based on PL spectra

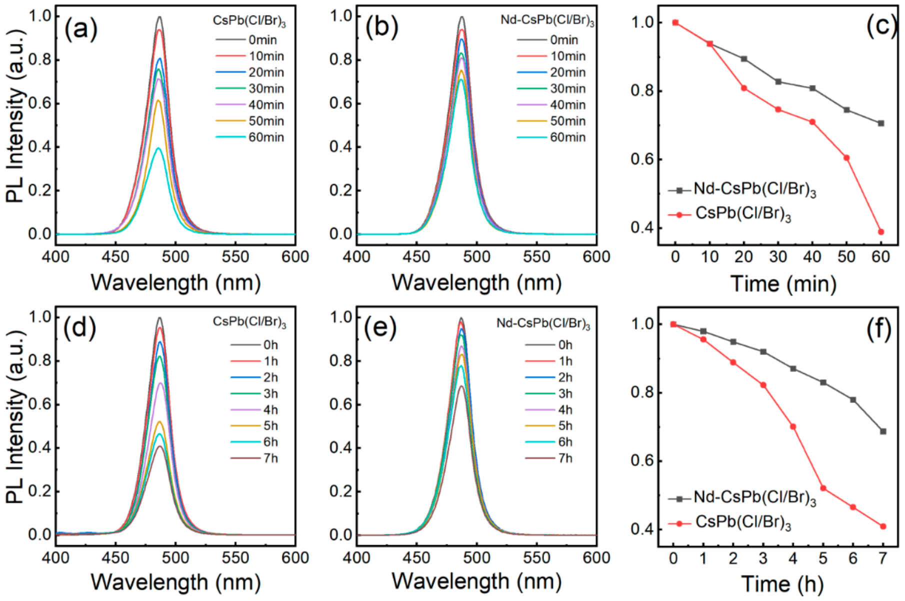

Figure S1. Effects of Nd-doping on (**a**-**c**) thermostability and (**d**-**f**) photostability of spin-coated films on glass slides. PL spectra and the relative intensity evolution over (**a**-**c**) a hotplate at 100℃ or (**d**-**f**) under continuous irradiation with 365 nm UV lamp.

S2: Effect of Nd doping on Exciton binding energy

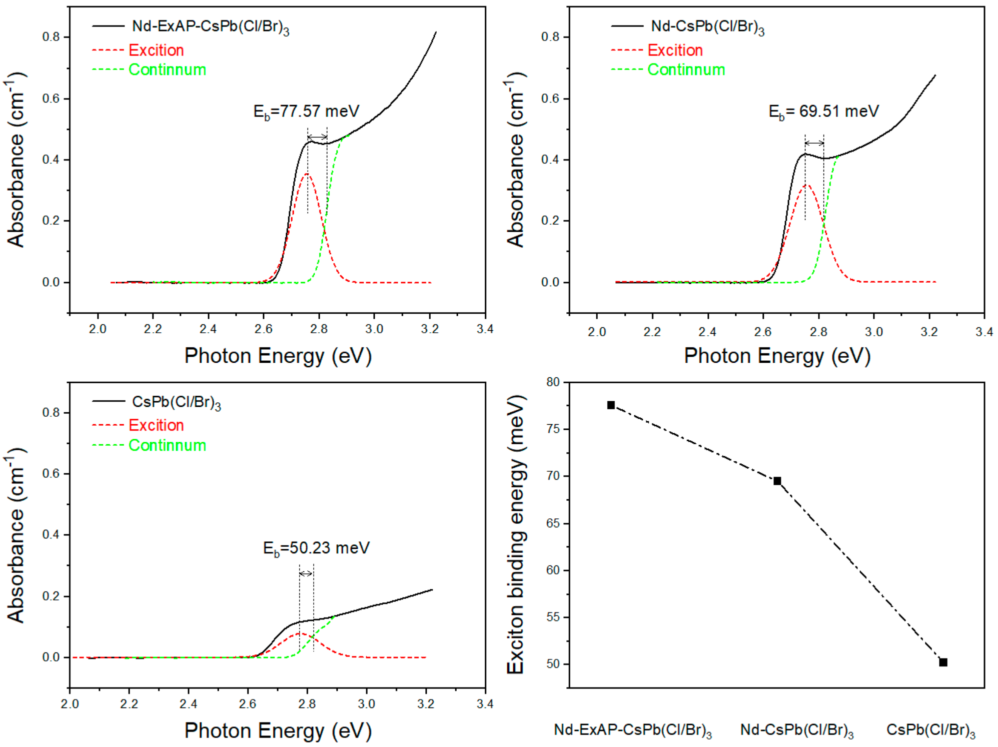

Figure S2. The exciton binding energy was calculated by the Elliotts model. The Nd doping would increase exciton binding energy.

S3：Effect of the BTBT mass fraction on emission wavelengths and CIE coordinates

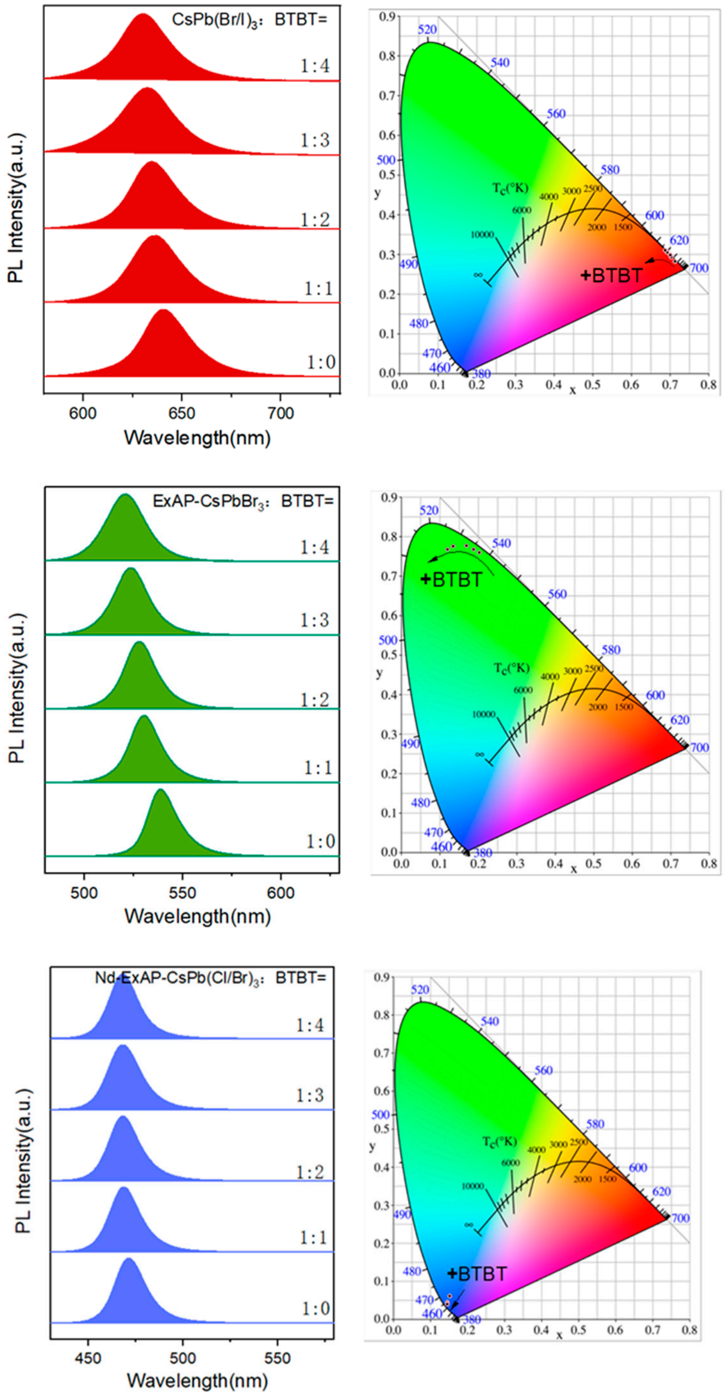

Figure S3. Effect of the amount of BTBT on the red shift. FRET can be eliminated by sufficient dilution.

S4: Effect of surface passivation based on PL spectroscopy on stability

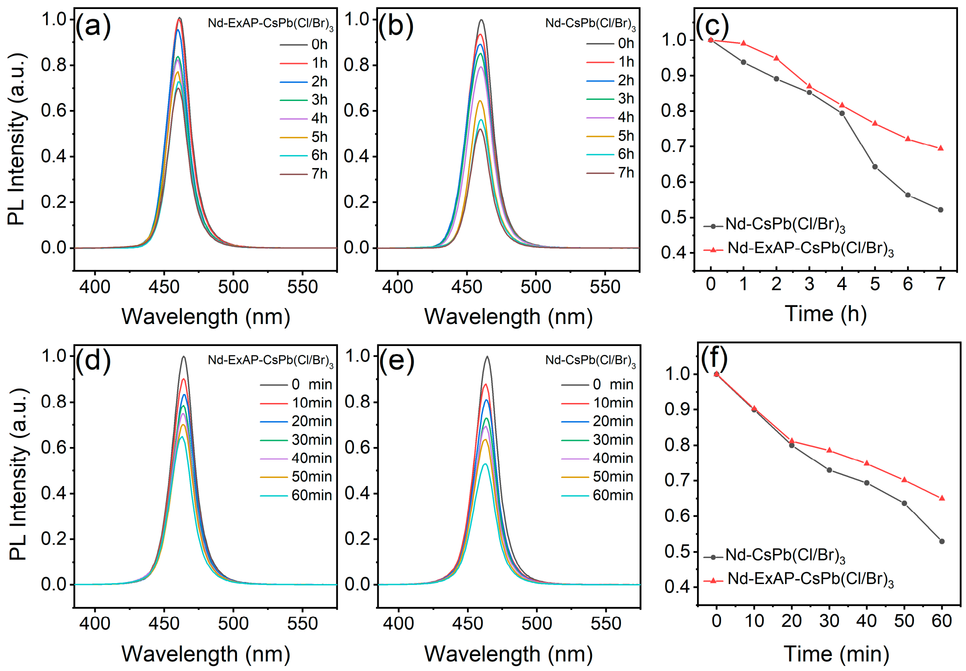

Figure S4. Effects of surface passivation on (**a**-**c**) Thermal and (**d**-**f**) Photo stability of spin-coated films on glass slides. PL spectra and relative intensity evolution (a-c) under 100 °C heating or (d-f) continuous irradiation with 365 nm UV lamp.

S5: Photostability of BTBT composite CCLs under 466 nm and 440 nm blue LED backlight.

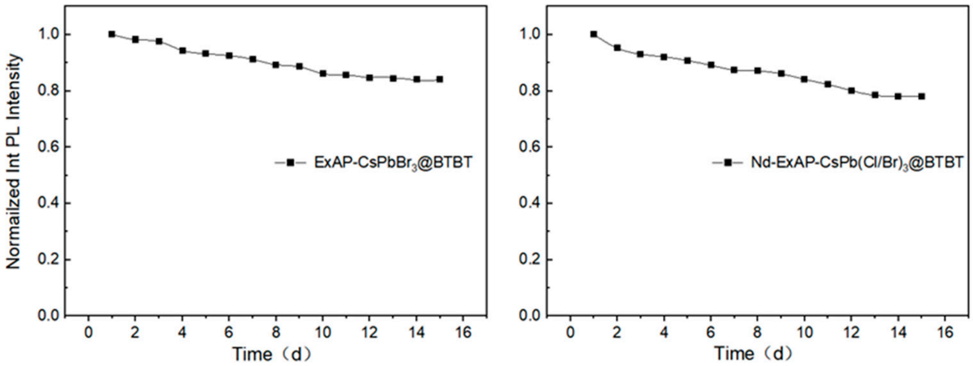

Figure S5. PL intensity of BTBT composite CCLs under prolonged exposure to blue LED backlight (in days), Left panel: green LED under 466 nm photoexcitation, Right panel: blue LED under 440 nm photoexcitation.
